# Whole-Genome Sequence of *Thermodesulfomicrobium* sp. Strain WS, Isolated from Onikobe Geothermal Field in Japan

**DOI:** 10.1128/mra.01321-22

**Published:** 2023-02-22

**Authors:** Md. Gahangir Alam, Shin Haruta

**Affiliations:** a Department of Biological Sciences, Tokyo Metropolitan University, Hachioji, Tokyo, Japan; Wellesley College

## Abstract

We present here the whole-genome sequence of a thermophilic chemolithotrophic sulfate-reducing bacterium, *Thermodesulfomicrobium* sp. strain WS (class Deltaproteobacteria), isolated from Onikobe geothermal field (Miyagi Prefecture, Japan). The genome consists of a 2,357,780-bp chromosome with a 62.9% GC content and 2,196 protein-coding sequences.

## ANNOUNCEMENT

Dissimilatory sulfate reduction is widely found in prokaryotes. However, thermophilic sulfate-reducing prokaryotes are limited in several phylogenetic lineages (1). Recently, a novel genus, *Thermodesulfomicrobium*, was proposed for thermophilic sulfate-reducing bacteria in the class Deltaproteobacteria (2). The type species was reported as Thermodesulfomicrobium thermophilum in 2007 (3), but only one strain, P6-2^T^, was isolated in the report, and the whole-genome sequence of this type strain is not yet available. To explore thermophilic sulfate-reducing bacteria, we anaerobically cultivated sediments collected at Onikobe geothermal field (38.48 N, 140.40 E; Miyagi Prefecture, Japan) at 55°C. A pure culture of strain WS was obtained after serial dilution to extinction and sequential transfers into agar shake vials and grown chemolithotrophically at 55°C (see below). A partial 16S rRNA gene sequence analysis, conducted using RDP SeqMatch (4), indicated that strain WS was classified in the genus *Thermodesulfomicrobium* (unpublished data). Here, we report the complete genome sequence of strain WS to contribute to evolutionary and physiological studies of sulfate-reducing bacteria.

Strain WS was anaerobically grown at 55°C in a medium (pH 7.5) containing (liter^−1^) 0.84 g NaNO_3_, 2.12 g Na_2_SO_4_, 0.75 g KH_2_PO_4_, 0.78 g K_2_HPO_4_, 4.2 g NaHCO_3_, 0.5 g cysteine HCl, 0.24 g Na_2_S·9H_2_O, 1 mL of a vitamin mixture (5), and 5 mL of a trace mineral solution (6) under a H_2_/CO_2_ (4:1, vol/vol) atmosphere. Genomic DNA was extracted using enzymatic lysis and purified using the Genomic-tip kit (Qiagen). A sequencing library was constructed using the SMRTbell Express template preparation kit v2.0 (PacBio) after DNA shearing (target length, approximately 10 to 15 kb) using the g-TUBE device (Covaris). The library was sequenced using a Sequel II system (PacBio), and a total of 27,097 high-fidelity reads with an average length of 8,061 bp were obtained via SMRT Link v10.1.0.119528 (PacBio). After quality filtering using Filtlong v0.2.0 (https://github.com/rrwick/Filtlong), the high-quality reads were assembled using Flye v2.9 (7). The generated contig was checked using Bandage v0.8.1 (8) and CheckM v1.14.5 (9). A single chromosome of 2,357,780 bp with 62.9% GC content was assembled, with a coverage of 93×. The obtained genomic sequence was annotated using the DFAST pipeline (10). Default parameters were used for all software analysis.

The genome of strain WS consists of 2,196 protein-coding sequences, 53 tRNAs, and 3 rRNA clusters. The 16S rRNA gene sequences were 99.2% identical to Thermodesulfomicrobium thermophilum P6-2^T^. The average nucleotide identity by BLAST (ANIb; Genetyx v15.1.1) of strain WS with mesophilic relatives in the genus *Desulfomicrobium* was lower than 70%, although overall, members in the genus *Desulfomicrobium* shared over 74% ANIb. These findings support the conclusion that strain WS belongs to the genus *Thermodesulfomicrobium*. The key genes for the autotrophic Wood-Ljungdahl pathway (CO dehydrogenase and formate dehydrogenase) were reported in Desulfomicrobium baculatum DSM 4028^T^ (GenBank accession number CP001629) (11), and homologous genes were detected in other *Desulfomicrobium* species (Desulfomicrobium orale, CP014230; Desulfomicrobium escambiense, AUAR01000001). However, no homologous genes were detected in the genome of strain WS by BLAST search. These findings clearly indicate a distant relationship between the mesophilic and thermophilic lineages. The genome of strain WS contains genes related to molybdenum-iron nitrogenase, *nifHI_1_I_2_DKENB*, in a contiguous gene cluster and also encodes a transcriptional activator, NifA, which is uncommon in anaerobic diazotrophs (12).

### Data availability.

The whole-genome sequence has been deposited at DDBJ/ENA/GenBank under the accession number AP027130. The BioSample and BioProject accession numbers are SAMD00564984 and PRJDB14907, respectively. The raw sequence reads are available in the DDBJ Sequence Read Archive under the accession number DRR424563.
